# Estimating Pavement Roughness by Fusing Color and Depth Data Obtained from an Inexpensive RGB-D Sensor

**DOI:** 10.3390/s19071655

**Published:** 2019-04-06

**Authors:** Ahmadreza Mahmoudzadeh, Amir Golroo, Mohammad R. Jahanshahi, Sayna Firoozi Yeganeh

**Affiliations:** 1 Zachry Department of Civil Engineering, Texas A&M University, College Station, TX 77843-3136, USA; 2 Department of Civil and Environment Engineering, Amirkabir University of Technology, Tehran, Iran, 424 Hafez Ave, Tehran 15875-4413, Iran; 3 Lyles School of Civil Engineering, Purdue University, West Lafayette, IN 47907, USA; jahansha@purdue.edu; 4 School of Civil Engineering, College of Engineering, University of Tehran, Tehran 15875-4413, Iran; Sayna.Firoozi@ut.ac.ir

**Keywords:** RGB-D sensor, depth data, Microsoft Kinect One (V2), Microsoft Kinect, visual sensing, time of flight sensor, 3D pavement surface reconstructing, outdoor imaging, pavement roughness, International Roughness Index (IRI), pavement condition assessment, pavement health monitoring

## Abstract

Measuring pavement roughness and detecting pavement surface defects are two of the most important tasks in pavement management. While existing pavement roughness measurement approaches are expensive, the primary aim of this paper is to use a cost-effective and sufficiently accurate RGB-D sensor to estimate the pavement roughness in the outdoor environment. An algorithm is proposed to process the RGB-D data and autonomously quantify the road roughness. To this end, the RGB-D sensor is calibrated and primary data for estimating the pavement roughness are collected. The collected depth frames and RGB images are registered to create the 3D road surfaces. We found that there is a significant correlation between the estimated International Roughness Index (IRI) using the RGB-D sensor and the manual measured IRI using rod and level. By considering the Power Spectral Density (PSD) analysis and the repeatability of measurement, the results show that the proposed solution can accurately estimate the different pavement roughness.

## 1. Introduction and Background

A pavement management system is a set of tools and methods that allow the authorities to determine optimum maintenance actions on pavement sections to enhance the pavement performance level. The pavement management system consists of different parts, and the most important part is data collection from pavement to examine its condition. According to Haas and Hudson [1], four major techniques that can be applied for comprehensive assessment of pavement conditions include pavement roughness, pavement defects, structural damage, and resistance to sliding (safety). Among all, pavement roughness is of significance due to ride quality and road safety.

According to an ASTM standard (Academic Society for Testing and Materials standards), pavement roughness is the vertical surface oscillations in pavement that reduces ride quality [2]. In other words, roughness is a change in the pavement alignment that shakes the vehicle and can be felt by a road user. Pavement roughness is measured between two points on the road by measuring the changes in the elevation level on the longitudinal profile (m/km or inch/mile) [2]. Since 1960, different indices were proposed to measure pavement roughness such as Mean Panel Rating (MPR), Profile Index (Ride Number), and International Roughness Index (IRI). The latter one is the most common, which was introduced initially by the World Bank in 1982, and it has been used widely since then [3]. This index provides an opportunity for obtaining the same values in different situations, from different vehicles or similar vehicles at different data collection periods. The response type measurement system is used to compute this index. First, the distance between a vehicle and the road surface, and the vertical displacement of the vehicle are measured. Then, vehicle motion on the road is modeled by applying various mathematical filters. Ultimately, the total cumulative displacement of the simulated spring system (as it is used in the car wheels) is divided by the traveled distance [4]. The IRI is affected by wavelengths of 1.2 to 30 meters. The index will be less than 0.5 (unit of slope) for the wave numbers of 0.033 $\frac{1}{m}$ and 0.8 $\frac{1}{m}$ corresponding to the wavelengths of 30 and 1.25 m, respectively.

**Figure 1:**
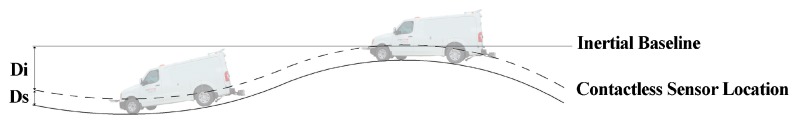
Basis of the inertial profiler.

Devices used in collecting pavement roughness data can be divided into three categories including profilers, profile graph, and response type systems. Profilers are indicating the precise pavement profile, which can be divided into three general categories of static, low-speed (inclinometer) and high-speed (lightweight and heavyweight) profilers. The most common one is a static profiler, such as a rod and level, which is used for measuring the base profile of a road and calibration of other devices due to its high accuracy [5]. The low-speed profiler records the slope by moving the profiler along the road, so this method has a higher data collection speed rather than the rod and level method. The high-speed profilers compute the roughness using the concept of the high-speed inertial profiler by creating an inertia baseline to compensate for the vertical displacement of the vehicle body. An accelerometer measures the vertical acceleration of the vehicle, and it is converted to the distance by double integration for finding the inertia baseline. Figure 1 illustrates the baseline and the pattern of a vehicle moving on a road. The distance between the ground and the baseline above can be used as elevation data for measuring the IRI. This distance is measured by adding the distance between the accelerometer to the ground (D_s_) and the distance between the accelerometer to the baseline (D_i_). A contactless sensor, such as ultrasonic, laser beam, or an optical image, measures the vertical distance between the car and the ground [5].

The profile graph is used manually at the walking speed by an operator, often to measure the longitudinal profile of concrete pavement. The last category applies response-type systems. This method measures the pavement profile by using cell phones or accelerometers mounted on a vehicle [6]. 

Due to the recent advances in science and technology, various companies operate in the competitive market to manufacture new profilers. A common characteristic of these profilers is the high cost, which is due to using laser technology. Under-developed or developing countries cannot afford such a device. Hence, it is necessary to search for devices with not only sufficient accuracy and precision in pavement data collection but also lower costs. RGB-D sensors are cost-effective devices that have not received enough attention for pavement data collection. The capability of an RGB-D sensor to capture the depth data and RGB images, as well as the low price of this product, make these sensors an appropriate device for measuring the pavement roughness. This study investigates the feasibility of using such a system for autonomous estimating of IRI when a data analysis procedure is also proposed. Moreover, the developed system is capable of creating a 3D-reconstruction of pavement surface, which can deliver a continuous longitudinal (as well as transverse) profile of each wheel path. These recorded data can be used later for assessment of various pavement conditions such as potholes, patching, and cracks [7].

## 2. Literature Review

The relevant studies can be categorized into pavement roughness indices, pavement roughness data collection methods, and the RGB-D sensors. Regarding the pavement roughness indices, Queiroz et al. proposed a roughness index for calibration of a quarter-car index [5]. Some studies developed theoretical indices considering the interactions between vehicles and roads. For instance, Liu and Herman (1998) examined the interplay between the road and user comfort [8]. In another study by the same authors, the effect of a jerk (bump) on a road as a response index was investigated [9]. Cantisani et al. showed that the IRI does not express the driving convenience by itself [10]. Other indices should be developed that consider the vertical acceleration and the driving convenience. Different studies have been performed on the computation of pavement roughness with regard to the factors affecting the vertical acceleration of a vehicle. Zhang et al. (2014) considered the Root Mean Square (RMS) of vertical acceleration as a measuring index of ride quality and pavement roughness. The results of this study were consistent with the ISO 2631 standard, which is a standard regarding human body exposure to the vibration [11]. Using a quarter-car model and considering 36 sections of a pavement surface, Sun (2002) associated the IRI to the Power Spectral Density (PSD) roughness utilizing a linear regression [12]. 

Researchers applied several pavement roughness data collection devices. Alhasan et al. (2015) examined the possibility of using a stationary laser for measuring IRI. Pointclouds taken from the surface via a fixed laser scanner were mapped side-by-side and analyzed with the Fourier transform [13]. This approach can be used on pavement that cannot tolerate the weight of profilers. Fernando et al. (2014) validated the measured IRI via Dynatest, which is an automated data collection vehicle, by using a rod and level on the same paths [14]. In another study, considering the wavelength of a profile as a roughness index of the pavement surface, the correlation between vehicle vibration and IRI values were measured by driving in a passenger car. The results show that, by having the same IRI values, there could be a difference of factor 30 between the best and worst ride, which can be used in making policy in the pavement management system (PMS) field. However, this method is not applicable for heavy vehicles [15]. Hesami et al. (2009) used signal processing for measuring pavement roughness. The profile data at different time intervals were collected and the energy level in wave bands at different altitude levels of road surface were estimated by using the PSD analysis. They showed that there is a good correlation of the calculated index with IRI [16]. In another study, road profile wavelengths were analyzed to measure pavement roughness. The high-frequency defects such as cracks and potholes were successfully identified. The results of the study showed that the wavelength analysis method could measure the pavement roughness with better accuracy than other signal processing methods [17].

The Laser Crack Measurement System (LCMS) that was 3D scans of the pavement profile at highway speed has made it possible to assess the IRI and other road surface characteristics [18]. Chang et al. (2009) computed the IRI inside a laboratory by using an automated robot equipped with a Coherent Change Detection (CCD) Laser and a laser range finder from the SICK sensor manufacturer. The computed IRI was validated by using the ARRB profiler [19]. This study did not consider the effect of speed or speed change on the proposed algorithm. In a study by Suksawat (2011), a low-cost measuring tool consists of five connected wheels, accelerometers, variable resistors, an encoder, and an angle sensor that was made for measuring the pavement roughness [20]. Zhang et al. (2015) computed the real-time pavement roughness using outputs of the tensile-compressive sensors embedded in the pavement. This measurement tool requires on-site installation and calibration, and the results showed that it could measure the IRI with high precision. However, to measure the IRI, the sensors need to be implemented close to each other (less than one meter distance) [21]. Yi and Ma (2009) introduced a new range finder with a symmetric but reverse structure. Pavement roughness was computed based on the non-inertial reference frame transfer by using five laser rangefinders mounted on a rod. The IRI was measured based on adjusting errors caused by a vehicle vibration, which are similar to the changes in the height of the longitudinal profile [22]. Recently, a pressure sensor was used for measuring pavement roughness [23]. Zhao (2015) measured pavement roughness by using directional microphone sensors and a dynamic tire pressure sensor with an axial accelerometer. The proposed method is validated for new pavement surfaces with the IRI of less than two, and it cannot distinguish the exact IRI greater than two [23]. 

Because these pavement evaluation methods are expensive and time consuming, smartphones have emerged as an inexpensive alternative. Smartphones are highly useful in data collection since they have sensors for recording the profile [24]. Douangphachanh and Oneyama (2013) developed an index similar to IRI by examining the correlation between speed and acceleration recorded by smartphones [25]. Using the “Androsensor” smartphone application, they installed two smartphones in the same direction in two different vehicles that traveled at almost constant (but different) speeds. The results showed that the type of vehicle affects the outputs, but different smartphones used in a vehicle did not lead to much difference in the outputs. Alessandroni et al. (2014) examined the possibility of computing the ride quality using data collected from the three-axis of an accelerometer. Using Linear Predictive Coding (LPC), they removed the accelerations data, which were not associated with surface roughness, such as vibrates. This results from gravity acceleration, vehicle acceleration, eccentricity, rotation, and going downhill [26,27]. However, the proposed method did not consider the effect of the vehicle suspension.

Another alternative to using smartphones in evaluating pavement conditions is the use of the Microsoft Kinect. The Kinect sensor was launched in two versions within four years by the Microsoft Corporation (Xbox 360 (2010) and One (2014)). The Microsoft Kinect One (Kinect V2) included an infrared laser emitter, an infrared ray absorber sensor, a Red-Green-Blue (RGB) sensor, a rotating motor on the base, and microphones to detect the external sound in different directions. The analytical features of Microsoft Kinect 1 and Microsoft Kinect Xbox 360 were presented in Mahmoudzadeh et al. (2015) [28]. Microsoft Kinect Xbox 360 cannot capture images in all environments since it provides low quality or incomplete depth images under direct sunlight. Thus, providing shade on the road surface is necessary when the sensor is used under direct sunlight. The infrared sensor is not capable of detecting infrared reflective rays from the surface under direct sunlight [29]. Another study has investigated the effect of different light intensities on the quality of data. The computed depth maps were compared in different light intensities, which were measured by an illuminance meter in lux. The results showed that the depth values were highly reliable under different light intensities and the light intensity did not have a significant impact on the quality of obtained depth data unless the sensor was exposed to direct sunlight [7]. However, the Microsoft Kinect Xbox 360 cannot work accurately in outside environments. The Microsoft Kinect One has better performance than Microsoft Kinect Xbox 360 in the outdoor environment, since it uses ToF technology [30,31,32]. A study by Fankhauser et al. confirmed that capturing data up to a one-meter distance from the surface under shadow is accurate, while errors are caused by increasing the range or changing the “incident angle of sunlight radiation” [33]. Another study analyzed the depth measurement accuracy for different illumination levels (measured based on Lux). They declared that increasing the illuminating intensity degrades the measured depth accuracy of a ToF camera [34].

In addition, a pixel might contain objects in different distances, which usually occur on edges. The return light from the foreground and background objects might generate another distance for the corresponding pixel. In other words, the difference in a solid angle causes a false phase shift due to the false distance information. This shift is called the flying pixel [35]. Some studies show that flying pixels occur in two situations: when an object is located under direct sunlight radiation and when there are two reflected pulses for each pixel [31,36]. However, by adding a shelter over the surface and taking pictures under the shadow, this problem may be solved. Regarding characteristics, Microsoft Kinect One has a depth image resolution of 512 × 424 pixels with 70.6 and 60 degrees horizontal and vertical fields of view, respectively. The color sensor resolution in the Kinect One is 1920 × 1080 pixels, with 84.1 and 53.8 degrees of horizontal and vertical fields of view, respectively. Microsoft Kinect One uses Time of Flight (ToF) technology, which uses a continuous laser wave in the form of a signal by considering radiation and reflection rays as two different phases. By measuring the time between sending and receiving the wave to/from an object, a 3D image can be reconstructed. By converting time to distance, the distance matrix is measured. Each cell of the matrix represents the distance between the object surface and the sensor, and the number of cells depends on the sensor imaging quality [32,37,38]. A few research studies have recently applied the Kinect sensor for the pavement condition data collection. Jahanshahi et al. (2012) used the Kinect Xbox 360 for detecting the pothole, patching, and crack on pavements. Since the collected data from Microsoft Kinect always has some noise, the measured depth surface is not parallel to the original surface [7,39]. Jahanshahi et al. used the median filter to reduce the noise and applied the RANSAC algorithm to enhance the measured depth surface and to make it parallel to the road surface. The accuracy of detection for the pothole, patching, and crack were 92%, 80%, and 78%, respectively [7]. Moazzam et al. (2013) used Microsoft Kinect Xbox 360 for capturing potholes. They measured the pothole area on asphalt and concrete surfaces. The results showed that the sensor has an accuracy of ±15% for measuring the pothole area [40]. Kamal et al. (2016) used Kinect to measure the depth and volume of potholes. They evaluated the Kinect performance in measuring the dimensions of potholes in different conditions (water versus oily water). The results showed that the mean percentage error of Kinect for measuring the depth is approximately 3% [41]. Chen et al. (2016) used multiple Kinect sensors to evaluate the road surface condition in a dynamic mode. Having solved the problems related to data collection at high speed, such as the motion blur problem and the rolling shutter distortion, they detected the surface defects by running the equipped vehicle at the traffic speed limit [42]. Zhang et al. (2018) detected and classified the intensity of different kinds of cracks by measuring their widths, lengths, and areas. He found that the Microsoft Kinect sensor can measure cracks with an acceptable accuracy [43].

In winding up the discussion, the scholars employed only Microsoft Kinect Xbox360 to detect some pavement surface defects such as potholes, patches, and cracks. However, the pavement roughness assessment using Microsoft Kinect One (Kinect V2) was not performed. 

## 3. Objective and Scope

This study aims to measure the pavement roughness through the application of an inexpensive RGB-D sensor with a reasonable level of accuracy and precision. The scope of this research is to utilize Microsoft Kinect One mounted on a cart to capture images in a static mode. The authors declare that the static data collection could be time-consuming, while the goal here is the feasibility check. Asphalt pavements were employed as the case study for pavement roughness data collection. 

## 4. Research Methodology

**Figure 2:**
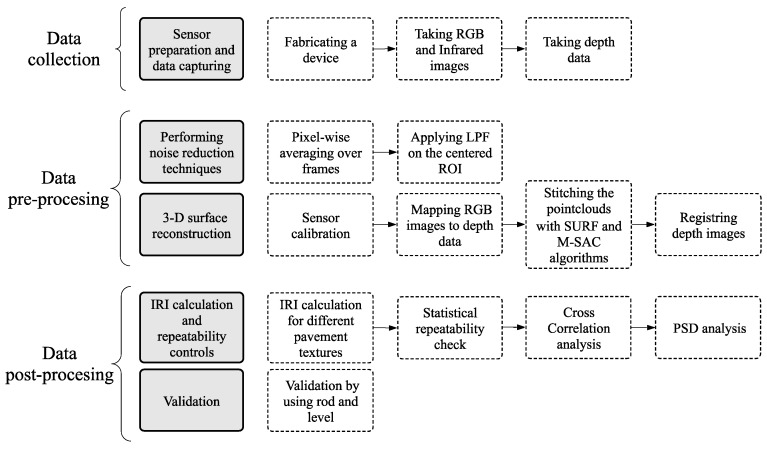
The methodology.

This research was conducted by collecting the data with a fabricated device (data collection), performing noise reduction and image matching techniques (data pre-processing), and computing IRI and validation (data post-processing). The steps are visualized in Figure 2 starting from the top to the bottom. It should be noted that the subtasks at each step are illustrated in hyphened rectangular shapes.

### 4.1. Data Collection

**Figure 3:**
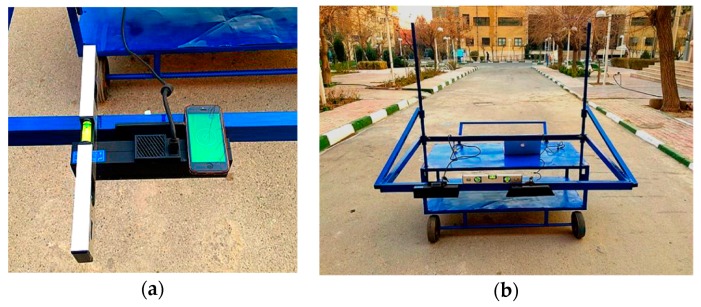
(**a**) Leveling the RGB-D sensor and (**b**) the data collection cart.

For collecting images from the pavement surface, it was essential to provide a distance between the sensor and the ground. For this purpose, a cart was designed and fabricated to be able to hold the sensor, as depicted in Figure 3. The dimensions of the designed cart were selected in such a way that, by installing two sensors on both sides, it would be possible to capture data over a 3.65 m width (i.e., a lane width). In addition, to create a suitable viewing angle for the sensor, an arm was added to the cart. 

The actual distance (ground truth) between the Kinect sensor and the surface was measured using a highly accurate laser meter. Then, 22000 depth values, which represent the distance between the Kinect and the reference surface, were extracted from a sample frame. The results show that the distance of the sensor from the surface affects the accuracy of the estimated depth data, since it was mentioned in previous studies [7]. Therefore, by testing different distances (from 0.8 m to 1.5 m), the results show that, by increasing the distance up to 1000 mm, an acceptable level of accuracy can be achieved. This distance is the minimum appropriate distance to reach an adequate coverage area between two consecutive images (according to the sensor field of view). From a different perspective, more coverage could be provided by tilting the camera. While the Kinect is capturing the vertical distances, it can be treated as higher dimension image capturing. 

In the process of data collection, two 50 m sections were chosen with different levels of roughness. Data was collected in the stationary mode by mounting the RGB-D sensor on the cart. The sensor was kept perpendicular to the ground, and it was controlled by using the engineering level in both vertical and horizontal directions (Figure 3a). However, it should be mentioned that it is not a necessary step in data collection. Two straight wheel paths were marked on the pavement, which were used to direct the wheels. From the beginning of the wheel path, stop points were marked at specified locations along each wheel path. The distance between each two successive stop points was similar. This distance satisfied the availability of the common features requirement when collecting successive images for the matching algorithm. The cart was moved along two marked wheel paths, and it was stopped at specified marked stations. After collecting 100 frames at a station, the cart was moved to the next station. Moreover, the wheels were locked in all directions except the straight one, at the current step of this study to follow the path lines. At each station, the RGB and infrared images were captured besides computing 100 frames of depth data on the pavement surface.

### 4.2. Data Pre-Processing

#### 4.2.1. Performing Noise Reduction Techniques

The depth data collected by the Kinect exhibits some noises, which should be reduced. The first step of this process was to smooth the depth data. Thus, the pixel-wise averages of frames were obtained to form the final depth matrix. In other words, each cell in the final matrix was computed by averaging the corresponding cells in the original frames. Moreover, a Gaussian filter was used as a low-pass filter to minimize the effect of small changes in the dataset. Furthermore, the averaging filter was used to remove the noise in the final depth matrix. 

**Figure 4:**
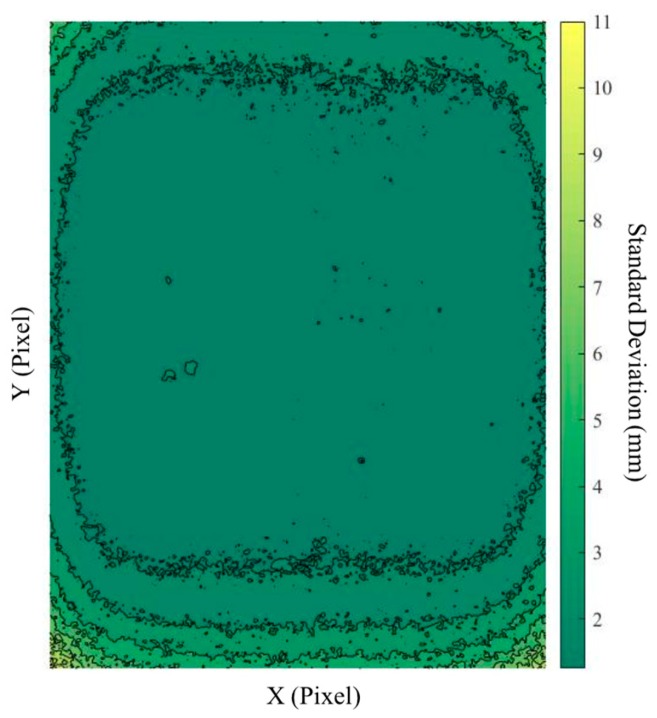
Standard deviation of each pixel over 500 depth frames captured from the same location.

In this study, to eliminate the noises at corners, the central part of the depth data (i.e., 450 × 350) was used for a further analysis. In Figure 4, the standard deviation of each pixel over 500 frames taken from a segment of the asphalt surface is plotted. The figure shows that, by moving toward the central part of the data matrix, the standard deviation of data is reduced. This is due to lens distortion of the IR camera that introduces some errors since the region of interest moves toward their borders.

#### 4.2.2. 3-D Surface Reconstruction

After preprocessing and noise removal, sensor calibration was performed. The sensor provides RGB image and depth data, which should be used to create the 3D surfaces. Calibration is needed to map the RGB image on the depth image, which will be used to stitch point clouds of the road surface, as explained later in this section. 

The sensor calibration consisted of two internal and external calibration steps. The “intrinsic parameters” were found by capturing images from a checkerboard using the Caltech toolbox for calibration [44]. Moreover, captured depth images were mapped with the RGB images because two separate sensors took the images. “External calibration” contained rotation and transmission parameters in the form of a transformation matrix between the RGB and the depth-imaging sensor. It should be mentioned that, due to the presence of such matrices, there is no need to put the camera perpendicular to the surface. 

**Figure 5:**
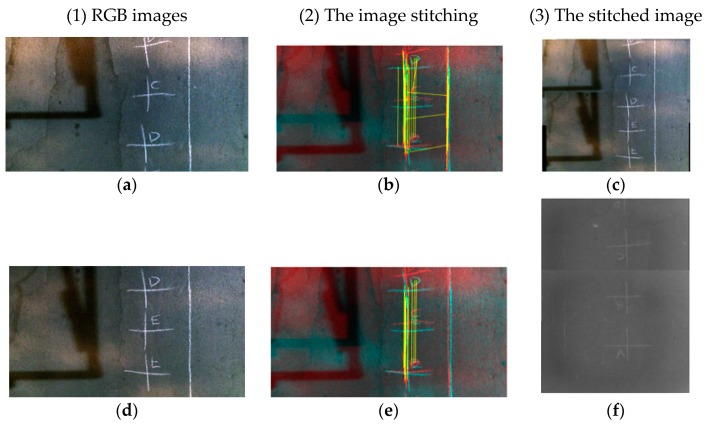
Creating a stitched image using a feature-based algorithm by having multiple images. (**a**,**d**) RGB images; (**b**,**e**) stitching process; (**c**,**f**) stitched images.

Since the IRI presented on a scale of 5, 10, or 100 m in length, it is necessary to match the depth matrices to create segments in such lengths. To this end, depth images were stitched together, as explained in this section, by using a feature based stitching algorithm (Figure 5). By using the SURF algorithm [45], the respective matching points of the two RGB images (Figure 5a,d) from adjacent sections of road were determined (Figure 5b,e). The white lines refer to Figure 5d and the red lines refer to Figure 5a. The yellow lines represent the matching features in the overlapping region of the two adjacent images. The M-estimator Sample Consensus (MSAC) algorithm, which is a subset of the Random Sample Consensus algorithm (RANSAC) [46], was used to eliminate the noise and error in detecting the corresponding points [47]. Figure 5c,f show the RGB and IR stitched images, respectively.

**Figure 6:**
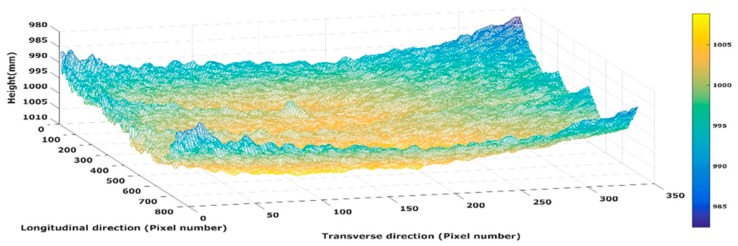
A 3D point cloud obtained by registering three consecutive depth images.

Once the transformation matrix between two RGB images is computed, since the transformation matrix between RGB and the depth sensor is already obtained from camera calibration, the depth images can be registered and stitched together. Figure 6 shows a 3D point cloud obtained by registering three consecutive depth images.

To sum up, this study focuses on developing an index using a longitudinal profile of a road segment. The most important task is to map/stitch images rather than localizing them on a map. On the contrary, in a full large-scale simultaneous localization and mapping method, images are captured with a position tag using GPS to be presented on a map [48]. The camera position is not important in this study because the aim was developing a road profile rather than presenting the pavement images on a map. Related literature shows that, by fusing the GPS and IMU data, location data with a higher frequency can be generated [42]. In addition, since a GPS dataset creates timestamps on captured images in a sequence, the images can be labeled with time and location [49]. It can also calculate the distances between images using latitude and longitude of different points, which were used in overlapping estimation [50,51].

### 4.3. Data Post-Processing

#### 4.3.1. IRI Calculation and Repeatability Controls

To compute IRI, 3D point clouds that constitute the longitudinal profile of each path were imported into ProVAL. ProVAL is approved by FHWA and applies the quarter-car method to compute IRI. The scale was computed by entering the resolution of the image (i.e., the distance of pixels from each other in the real world). To evaluate the precision of measuring IRI, two different methods are used. First, data are collected for five times for each of four segments to assess the statistical difference between replicates regarding calculated IRI values. Then, the cross-correlation analysis is used to evaluate the spatial dispersion of data over the length of segments. Moreover, the Power Spectral Density is calculated to analyze the dispersion of wavelength in the dataset, which gives a better sense of the roughness dispersion over the length of a segment instead of calculating a single number for defining roughness.

#### 4.3.2. Validation

**Figure 7:**
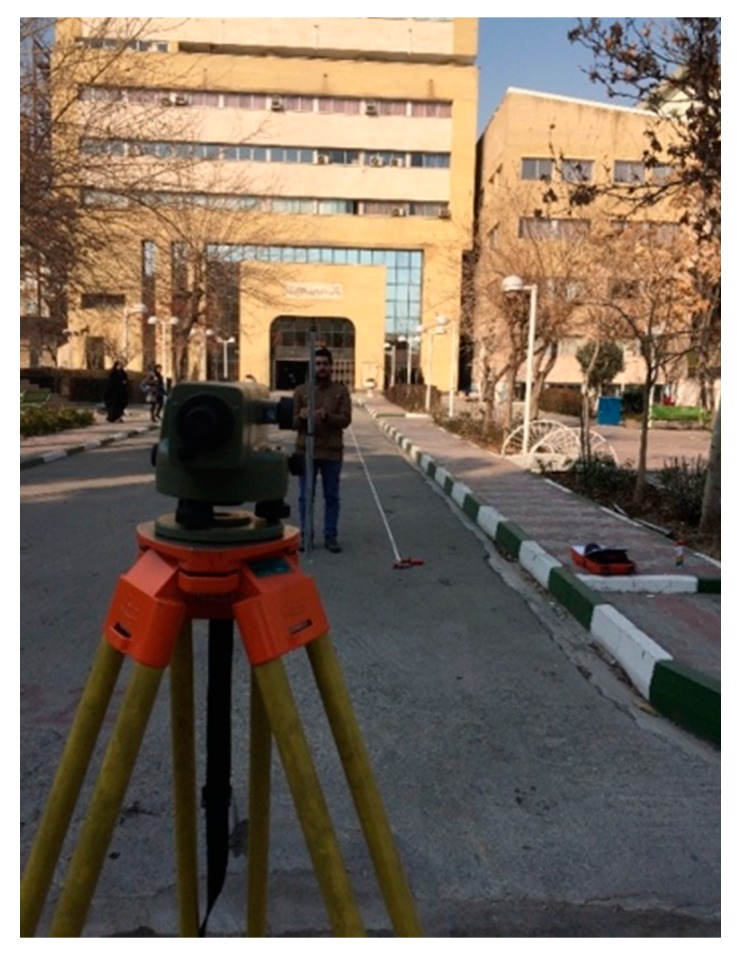
Data collection with the rod and level for the validation purpose.

Lastly, it is necessary to re-calculate the IRI by rod and level to validate the sensor outputs (Figure 7). For this purpose, the same road sections used for data collection were divided into 30-cm intervals. The ASTM E1364-95 says that the maximum interval between data points should be less than 305 mm (1 ft) for the Class 1 resolution [52]. Moreover, the ASTM E 1926-08 allows the researchers to choose an interval based on their opinion, and it suggests using an interval of 0.3 m (12 in) or less. However, reducing the interval typically improves the precision [3]. Moreover, the sample interval for collecting the IRI via rod and level was 30 cm, and the length of 10 m for each path with at least 33 data points were used to calculate an IRI for each path. Twenty paths were considered to evaluate the validity of this study, which is more than enough data points for evaluation. These were used from the statistical point of view. Moreover, 20 points of data (20 values for IRI) is enough for a population, which is not severely skewed.

The data collection technique should meet the criteria mentioned in ASTM 1364-95 [52]. According to ASTM 1364-95, if the rod and level is used to validate the profilers (Kinect sensor), the techniques examined between two systems should be the same. Thus, in this study, the depth data was analyzed using ProVAL software.

This section may be divided by subheadings. It should provide a concise and precise description of the experimental results, their interpretation, and the experimental conclusions that can be drawn.

## 5. Results and Discussions

**Figure 8:**
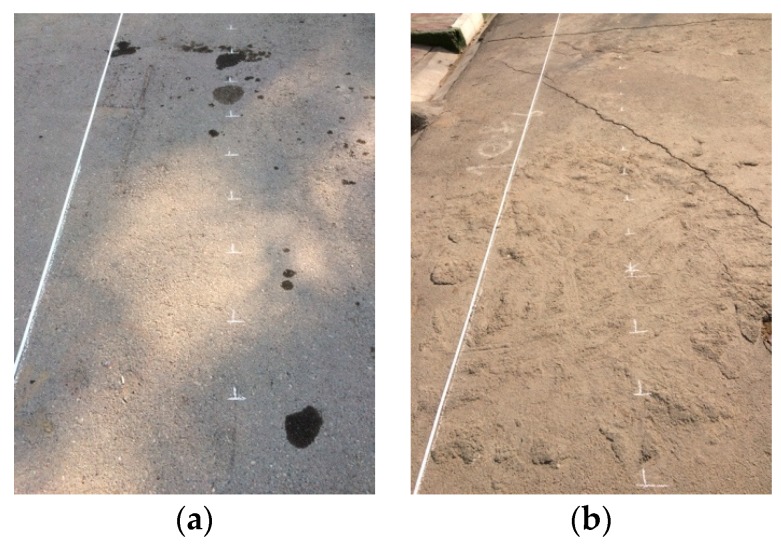
Sample surfaces used for evaluation: (**a**) smooth surface and (**b**) rough surface.

In this section, the results of computing the IRI of six 10-m road segments are discussed. As previously mentioned, two sections with the length above 50 m for each, were selected with one smooth surface (Figure 8a) and one rough surface (Figure 8b). This section follows the order mentioned in the data post-processing section of the flowchart, by discussing the statistics behind the IRI values, the repeatability controls, the cross-correlation analysis, the PSD analysis, and the validation results, respectively.

### 5.1. IRI Calculation for Different Pavement Textures

IRI values were categorized into intervals in length of two units [3]. Table 1 shows the number of segments investigated at each interval, mean, standard deviation, standard error, confidence interval, and minimum and maximum values of data at each interval. This table shows that 48 segments have an IRI between 2 (m/km) and 4 (m/km), while the average IRI is 3.29 (m/km). In addition, it shows that the overall dispersion of the pavement roughness indices is between 2 (m/km) and 10 (m/km). In other words, the segments were covering a large range of IRI. Although there have been two roadway sections that were evaluated in Table 2, the results are reported in three IRI intervals based on Reference [5]. The adjustment is emphasizing the practical assessment of IRI, since the IRI thresholds in this table are defined based on practitioners’ standpoints.

**Figure 9:**
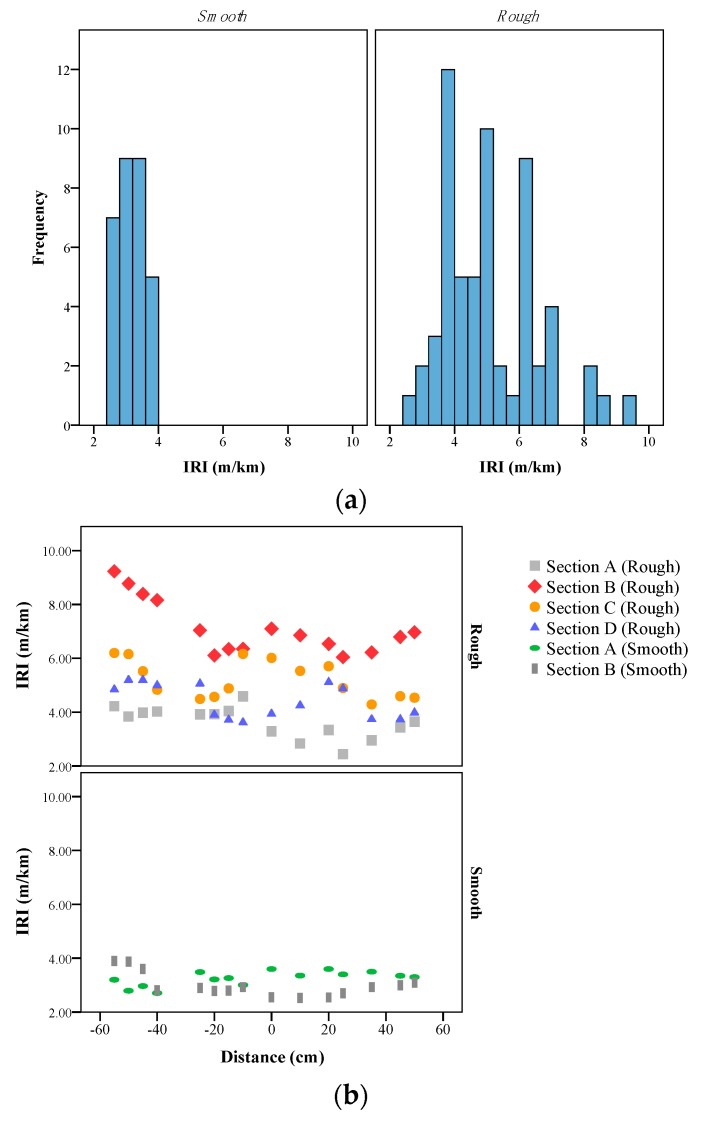
(**a**) Frequency of the collected IRI values in different sections and (**b**) the IRI values of each 10-m segments considering their location on each section.

The dispersion of the IRI values is shown in Figure 9a for different pavement conditions. In this study, 90 paths were analyzed, which were collected from two different surfaces. The smooth pavement had smooth paths, and the other one had rough pavement segments. The reason for selecting different surfaces was to show that the RGB-D sensor could be used in computing a broad range of pavement roughness indices. Figure 9a shows that the number of paths used on the rough surface was higher than the smooth surface. In this study, road sections were divided into 10-m segments and IRI values were computed in different segments.

**Figure 10:**
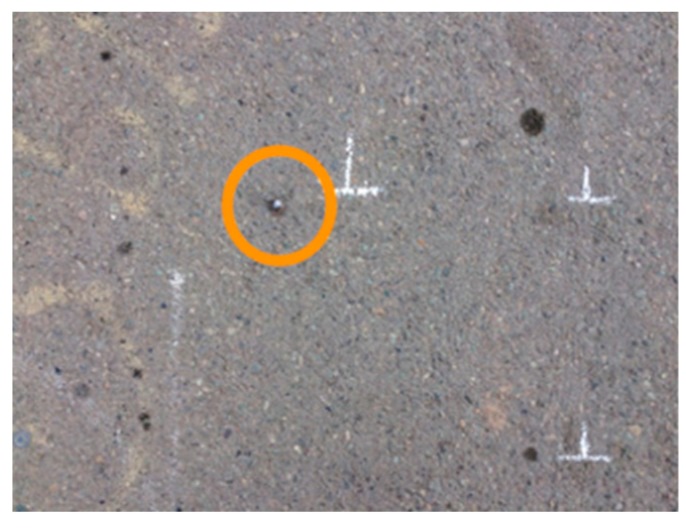
Sample external objects on the road: nail.

Figure 9b shows the IRI values of different segments. Four segments of the rough surface and two segments of the smooth surface with their corresponding computed IRI indices are shown in this figure. Each point in this figure indicates a computed IRI value, and each color indicates a segment. The horizontal axis in Figure 9b indicates the distance between the studied paths, where the reference was the path on the middle of the depth frame. This figure shows that, for the smooth surface, the average IRI values in different paths were closer to each other, such that, in 30 paths examined on the smooth surface, the dispersion of IRI was lower than 0.5 units. The results show that the IRI value of a segment cannot be found by measuring only two paths on each segment. Figure 9b shows that, in some segments, the difference between the minimum and maximum amount of IRI is more than two units. The red points from the rough surface confirm this claim. This difference might be due to the presence of some external objects on the road, such as nails (Figure 10) and the non-systematic error of the device. For instance, the presence of the shadow in the occluded areas might be a reason for this phenomenon [53].

### 5.2. Statistical Repeatability Controls

The repeatability of measuring the IRI is a requirement for developing an autonomous pavement roughness measuring system. Data collection from the rough surface was performed five times to examine the repeatability of computing the IRI using the RGB-D sensor. Table 3 expresses the descriptive statistics of five replicates on four segments (each one is 10 m). The mean and standard deviation of five replicates for each segment are given in Table 3. Additionally, the results of the ANOVA test among replicates, which are shown in each line, are given for the segments (df = 4). Table 3 shows that there is no significant difference between the replications since the level of significance of the ANOVA tests for all of the segments are higher than 0.05. The F test confirms it since the f statistics is greater than the critical value of F. In addition, it was found that the coefficient of variation in all four replicates was low (i.e., lower than 0.2), which confirms the precision of replicates on each segment.

### 5.3. Cross-Correlation Analysis

The Cross-Correlation method (CC), as a method for examining the repeatability and verification of pavement roughness measurement [54], was used in the study since this index is much more rigorous than when comparing the IRI values [55]. This method considers the spatial distribution of the roughness. The output of 100% in this method means that two depth data series are completely correlated, which can be treated as two signal waves. After selecting a replicate as the basis, the remaining ones were selected as replicates under repeatability testing. Table 4 shows the correlation between five depth signals, which represents the high similarity among these replicates. The second row in the table shows the calculated IRI in each replicate, the mean of all which is 3.92 (m/km). In the third row, the error (in percentage) in measuring the IRI values is shown, which was calculated by considering the IRI value of each run and the mean IRI value (3.92 m/km). This row shows that there is little difference between the calculated indices between different replicates. The fourth row shows the correlation between the basis signal and each replicate signal, which shows that there is a high correlation between replications, and all the correlations are above 90%. 

It should be mentioned that the cross-correlation shows how the two profiles are correlated with each other (i.e., how the roughness values along the two profile are correlated). Based on FHWA, the cross-correlation can be used to compare the magnitude and the spatial distribution of roughness over a segment. The overall IRI shows the overall roughness level along each segment [56]. Therefore, these two indices are two distinct measures, and they do not contradict each other. Therefore, it is possible that, while the overall IRI values are not close, the cross-correlation between the IRI values would be high [55]. 

**Figure 11:**
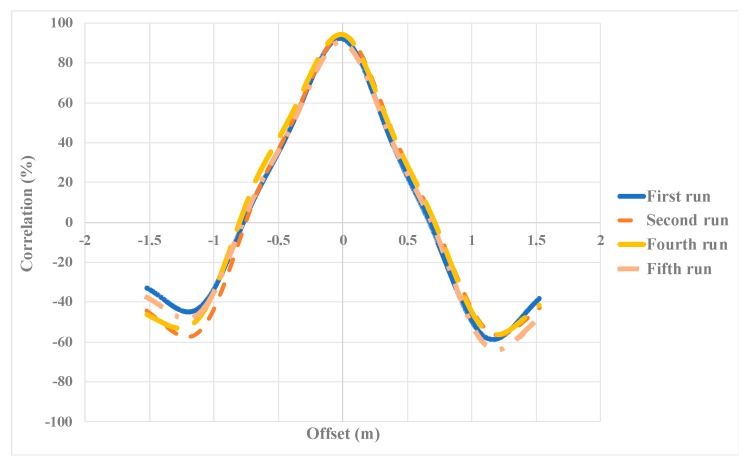
Cross-correlation analysis on depth data of a path for different runs.

Figure 11 shows the correlation diagram of the data series by choosing the offset, which illustrates that the optimal offset is very close to zero (lower than -0.02 in all replicates), which indicates the high similarity of the collected data series.

### 5.4. Study of Power Spectral Density (PSD)

It is also possible to measure the pavement roughness based on the wavelengths. The depth data collected from the road surface can be converted into several waves with different ranges using a Fourier transform. In the examination of two road paths, each might have the same overall roughness index, but with different PSD values at different intervals, which are related to the distributions of wavelength in the dataset. In this study, the PSD on the slope was used, which is more common in pavement engineering. The wavelengths lower than 3 m represent the abnormal phenomena in the upper layers of pavement, and wavelengths higher than 10 m are associated with abnormal states in the lower layers of pavement [57]. The PSD index has better functioning in longer paths, but, in this study, to evaluate the functionality of the primary design (i.e., using of the RGB-D sensor for evaluating the pavement roughness), the PSD index was utilized. 

**Figure 12:**
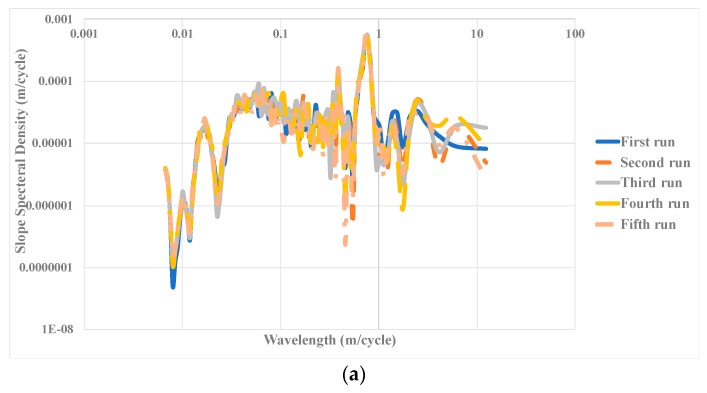

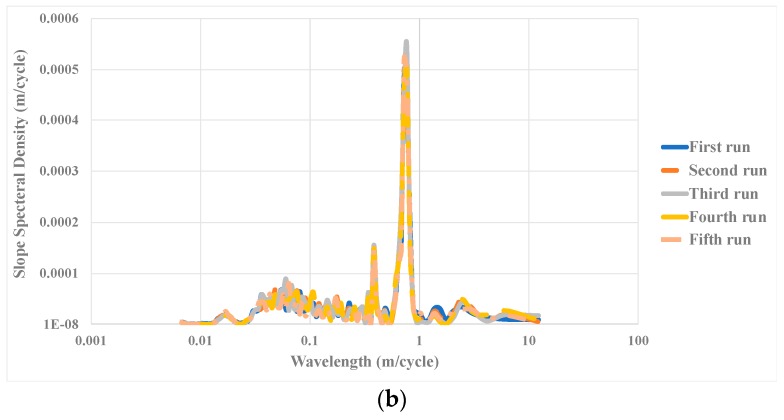
Slope spectral density based on: (**a**) wavelength and: (**b**) logarithm of the function.

Figure 12 shows the PSD values for different wavelengths. A segment from the rough surface with the highest dispersion and highest average IRI was chosen to determine the most sensitive wavelength of the collected data for a path. By changing the y-axis of Figure 12a to the logarithmic scale, it was found that, at a wavelength close to 1 m, there is a different pattern on the pavement surface (Figure 12b). This pattern is related to the upper layer of the pavement that can be associated with particles found on the pavement. This pattern might be based on the presence of some external particles on the pavement. This figure shows that the developed tool is capable of developing meaningful PSD values.

### 5.5. Validation

**Figure 13:**
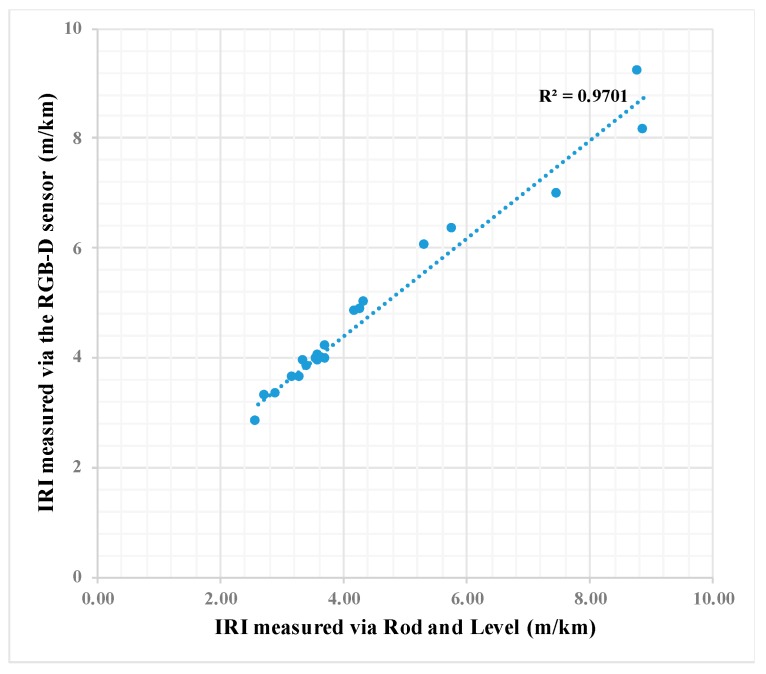
Correlation between IRI measured via rod and level, and the RGB-D sensor.

Data was collected for 20 paths from the 90 analyzed paths based on ASTM 1364-95 by using the rod and level, which is the best tool to find the reference roughness for a limited number of paths [52]. Figure 13 shows that there is a high correlation between the IRI measured by using the rod and level and the values estimated by using the RGB-D sensor (R2 is above 0.95). Moreover, by computing the difference between the IRI measured by the proposed approach, and rod and level, the measurement accuracy of 90% was achieved. The result of the Wilcoxon test shows that there is no significant difference between the values of IRI calculated by the two devices (Sig = 0.006, Z = -2.72). 

## 6. Future Work

Multiple directions are defined to continue this research. In terms of the pavement roughness measurement, since Kinect One can collect RGB-D data at the rate of 30 fps, the feasibility of computing IRI in motion need to be investigated even though the feasibility and data collection with traffic speed has been studied recently [42]. Moreover, a commercial automated data collection vehicle should be used to further evaluate the performance of the proposed approach in this study by comparing the IRI values obtained from a laser system and the proposed approach.

In terms of the algorithm, using more than one RGB-D sensor will help create a 3D road surface that covers the full lane width. From a different approach, using a sensor fusion of RGB-D cameras and odometry should be investigated [58]. This is proposed to collect the data on segments with more than 50 meters to check whether the error drift affects the computed pavement roughness. 

## 7. Conclusions

Measuring pavement roughness and detecting pavement surface defects is interesting to pavement engineers. To accomplish these tasks, managing the data collection as a core of the pavement management system should be performed. Data collection is very costly for authorities. Hence, it is important to look into expensive automated or semi-automated data collection approaches.

The main contribution of this study is to investigate the feasibility of using an inexpensive RGB-D sensor with an appropriate accuracy to measure pavement roughness. Such inexpensive sensors have not been used to compute the pavement roughness up to this point. RGB, infrared, and depth data are collected from different surface types (smooth and rough) with the different roughness levels at 10-m sections. By performing pixel-wise averaging over the collected frames at each imaging station, a single depth frame is calculated for each station. The noise reduction techniques are applied to each depth frame, the RGB images are mapped onto depth images, and the RGB and depth images are registered to create a 3D surface on the pavement surface. 

The complete 3D surface is divided into 90 paths, which are evaluated as multiple wheel paths. The paths are evaluated to obtain IRI. Ninety different paths are recorded with IRI values of 2 (m/km) to 10 (m/km) where 48 paths have an IRI value lower than four. In addition to IRI, PSD is measured and repeatability of data collection by an RGB-D sensor is assessed via the statistical methods and Cross-Correlation method. Lastly, by comparing the measured IRI valued via Rod & Level and the RGB-D sensor for 20 paths, it is found that the RGB-D sensor has a precision of 90%.

## Figures and Tables

**Table 1:** Descriptive statistics of the collected pavement.

IRI Values (m/km)	Frequency	Average (m/km)	Standard Deviation (m/km)	Standard Error	95% Confidence Interval	Min IRI (m/km)	Max IRI (m/km)
2–4	48	3.29	0.45	0.066	3.15–3.42	2.44	3.98
4–6	23	4.79	0.47	0.097	4.59–4.99	4.02	5.7
6–8	15	6.45	0.39	0.1	6.24–6.67	6.01	7.1
8–10	4	8.63	0.23	0.23	7.89–9.38	8.16	9.23

**Table 2:** Descriptive statistics of the collected pavement based on the pavement types.

IRI Values (m/km)	Pavement Type	Frequency	Average (m/km)	Standard Deviation (m/km)	Standard Error	95% Confidence Interval	Min IRI (m/km)	Max IRI (m/km)
**1.5–3.5**	**New pavements**	31	3.01	0.31	0.055	2.9–3.13	2.44	3.5
**2.5-6**	**Older pavement**	70	3.79	0.83	0.099	3.59–3.99	2.53	5.70
**3.5-10**	**Maintained unpaved**	60	5.16	1.42	0.183	4.79–5.52	3.5	9.23

**Table 3:** Repeatability of the records of the same path with ANOVA.

Segments	Average of IRI (SD) (m/km)	CoV	Sum of Square	Mean Square (Between Groups)	Significance	F.
**First**	3.63 (0.144)	0.040	0.02	0.005	1	0.15
**Second**	7.12 (0.225)	0.032	0.032	0.008	1	0.007
**Third**	5.22 (0.203)	0.039	0.02	0.005	1	0.009
**Fourth**	4.41 (0.235)	0.053	0.520	0.130	0.880	0.295

**Table 4:** Repeatability of the records of the same path with cross-correlation.

	First Run	Second Run	Third Run	Fourth Run	Fifth Run	Average
**IRI (m/km)**	3.9	4.08	3.92	3.93	3.77	3.92
**Error in measuring the IRI between each run and the mean (%)**	0.51	4.08	0.00	0.26	3.83	1.73
**Cross-correlation between each run and the basis run (%)**	92.5	94.2	-	94.2	90.5	92.85

## References

[1] Haas R., Hudson W.R., Falls L.C. (2015). Pavement Asset Management.

[2] (2012). ASTM International E867-Standard Terminology Relating to Vehicle-Pavement Systems.

[3] (2015). ASTM International ASTM E1926-08 Standard Practice for Computing International Roughness Index of Roads from Longitudinal Profile Measurements.

[4] Sayers M.W. (1995). On the calculation of International Roughness Index from longitudinal road profile. Transp. Res. Rec..

[5] Sayers M.W., Karamihas S.M. (1998). The Little Book of Profiling. https://trid.trb.org/view/1164606.

[6] Tighe S.L., Ningyuan L., Kazmierowski T. (2008). Evaluation of semiautomated and automated pavement distress collection for network-level pavement management. Transp. Res. Rec..

[7] Jahanshahi M.R., Jazizadeh F., Masri S.F., Becerik-Gerber B. (2013). Unsupervised approach for autonomous pavement-defect detection and quantification using an inexpensive depth sensor. J. Comput. Civ. Eng..

[8] Liu C., Herman R. (1998). Road profiles, vehicle dynamics, and human judgment of serviceability of roads: Spectral frequency domain analysis. J. Transp. Eng..

[9] Liu C., Herman R. (1999). Road profile, vehicle dynamics, and ride quality rating. J. Transp. Eng..

[10] Cantisani G., Loprencipe G. (2010). Road roughness and whole body vibration: Evaluation tools and comfort limits. J. Transp. Eng..

[11] Zhang Z.T., Zhao Q.M., Yang W.Q. (2014). Pavement roughness indices related to riding comfort. Appl. Mech. Mater..

[12] Sun L. (2003). Simulation of pavement roughness and IRI based on power spectral density. Math. Comput. Simul..

[13] Alhasan A., White D.J., De Brabanter K. (2017). Spatial pavement roughness from stationary laser scanning. Int. J. Pavement Eng..

[14] Fernando E.G., Walker R.S., Mikhail M. (2014). Comparative testing of lasers for ride quality measurement on hot-mix asphalt pavements. Transp. Res. Rec..

[15] Ahlin K., Granlund N.O.J. (2002). Relating road roughness and vehicle speeds to human whole body vibration and exposure limits. Int. J. Pavement Eng..

[16] Hesami R., McManus K.J., Evans R.P., Hassan R. A comparative study of roughness indices for monitoring the performance of thin seal flexible pavements subjected to low traffic volumes in Australia. Proceedings of the Civil, Structural and Environmental Engineering Computing.

[17] Hesami R., McManus K.J. Signal processing approach to road roughness analysis and measurement. Proceedings of the TENCON.

[18] Laurent J., Savard Y., Lefebvre D. 3D laser road profiling for the automated survey of road surface conditions and geometry. Proceedings of the 17th International Road Federation World Meeting.

[19] Chang J., Su Y., Huang T., Kang S., Hsieh S. Measurement of the International Roughness Index (IRI) using an autonomous robot (P3-AT). Proceedings of the 26th International Symposium on Automation and Robotics in Construction.

[20] Suksawat B. Development of multifunction international roughness index and profile measuring device. Proceedings of the 11th International Conference on Control, Automation and Systems.

[21] Zhang Z., Deng F., Huang Y., Bridgelall R. (2015). Road roughness evaluation using in-pavement strain sensors. Smart Mater. Struct..

[22] Zhang Y., Ma R.G. A study of pavement roughness measurement system based on laser ranger finder. Proceedings of the International Conference on Image Analysis and Signal Processing.

[23] Zhao Y. (2015). Road Condition and Road Roughness Assessment by Tire/Road Interaction Using Microphone, Dynamic Tire Pressure Sensor with an Axle Accelerometer.

[24] Islam S., Buttlar W.G., Aldunate R.G., Vavrik W.R. (2014). Use of cellphone application to measure pavement roughness. Proceedings of the T&DI Congress: Planes, Trains, and Automobiles.

[25] Douangphachanh V., Oneyama H. (2014). A study on the use of smartphones under realistic settings to estimate road roughness condition. EURASIP J. Wirel. Commun. Netw..

[26] Douangphachanh V., Oneyama H. Estimation of road roughness condition from smartphones under realistic settings. Proceedings of the 13th International Conference on ITS Telecommunications (ITST).

[27] Yeganeh S.F., Mahmoudzadeh A., Azizpour M.A., Golroo A. (2019). Validation of smartphone based pavement roughness measures. arXiv.

[28] Mahmoudzadeh A., Yeganeh S.F., Golroo A. (2015). Kinect, a novel cutting edge tool in pavement data collection. Int. Arch. Photogramm. Remote Sens. Spat. Inf. Sci..

[29] Mankoff K.D., Russo T.A. (2013). The Kinect: A low-cost, high-resolution, short-range 3D camera. Earth Surf. Process. Landf..

[30] Zennaro S. (2014). Evaluation of Microsoft Kinect 360 and Microsoft Kinect One for Robotics and Computer Vision Applications, Università di Padova. http://tesi.cab.unipd.it/47172/.

[31] Butkiewicz T. Low-cost coastal mapping using Kinect v2 time-of-flight cameras. Proceedings of the 2014 Oceans–St. John’s.

[32] Lachat E., Macher H., Mittet M.-A., Landes T., Grussenmeyer P. (2015). First experiences with kinect V2 sensor for close range 3D modelling. Int. Arch. Photogramm. Remote Sens. Spat. Inf. Sci..

[33] Fankhauser P., Bloesch M., Rodriguez D., Kaestner R., Hutter M., Siegwart R. Kinect v2 for mobile robot navigation: Evaluation and modeling. Proceedings of the International Conference on Advanced Robotics (ICAR).

[34] Achar S., Bartels J.R., Whittaker W.L., Kutulakos K.N., Narasimhan S.G. (2017). Epipolar time-of-flight imaging. ACM Trans. Graph..

[35] Lindner M., Schiller I., Kolb A., Koch R. (2010). Time-of-Flight sensor calibration for accurate range sensing. Comput. Vis. Image Underst..

[36] Breuer T., Bodensteiner C., Arens M. Low-cost commodity depth sensor comparison and accuracy analysis. Proceedings of the SPIE Electro-Optical Remote Sensing, Photonic Technologies, and Applications VIII; and Military Applications in Hyperspectral Imaging and High Spatial Resolution Sensing II.

[37] Chow J.C.K., Ang K.D., Lichti D.D., Teskey W.F. (2012). Performance analysis of a low-cost triangulation-based 3D camera: Microsoft Kinect system. Int. Arch. Photogramm. Remote Sens. Spat. Inf. Sci..

[38] Stoyanov T., Mojtahedzadeh R., Andreasson H., Lilienthal A.J. (2013). Comparative evaluation of range sensor accuracy for indoor mobile robotics and automated logistics applications. Robot. Auton. Syst..

[39] Jahanshahi M.R., Masri S.F., Padgett C.W., Sukhatme G.S. (2013). An innovative methodology for detection and quantification of cracks through incorporation of depth perception. Mach. Vis. Appl..

[40] Moazzam I., Kamal K., Mathavan S., Usman S., Rahman M. Metrology and visualization of potholes using the microsoft kinect sensor. Proceedings of the 16th International IEEE Conference on Intelligent Transportation Systems.

[41] Kamal K., Mathavan S., Zafar T., Moazzam I., Ali A., Ahmad S.U., Rahman M. (2018). Performance assessment of Kinect as a sensor for pothole imaging and metrology. Int. J. Pavement Eng..

[42] Chen Y.L., Jahanshahi M.R., Manjunatha P., Gan W.P., Abdelbarr M., Masri S.F., Becerik-Gerber B., Caffrey J.P. (2016). Inexpensive multimodal sensor fusion system for autonomous data acquisition of road surface conditions. IEEE Sens. J..

[43] Zhang Y., Chen C., Wu Q., Lu Q., Zhang S., Zhang G., Yang Y. (2018). A Kinect-Based Approach for 3D Pavement Surface Reconstruction and Cracking Recognition. IEEE Trans. Intell. Transp. Syst..

[44] Camera Calibration Toolbox for Matlab. http://www.vision.caltech.edu/bouguetj/calib_doc/.

[45] Bay H., Ess A. (2008). Speeded-Up Robust Features (SURF). Comput. Vis. Image Underst..

[46] Brown M., Lowe D. (2007). Automatic panoramic stitching using invariant features. Int. J. Comput. Vis..

[47] Torr P.H.S., Zisserman A. (2000). MLESAC: A new robust estimator with application to estimating image geometry. Comput. Vis. Image Underst..

[48] Scaramuzza D., Cadena C., Leonard J.J., Carrillo H., Latif Y., Reid I., Neira J., Carlone L. (2016). Past, present, and future of simultaneous localization and mapping: Toward the robust-perception age. IEEE Trans. Robot..

[49] Dong N., Ren X., Sun M., Jiang C., Zheng H. Fast stereo aerial image construction and measurement for emergency rescue. Proceedings of the International Conference on Geo-Information Technologies for Natural Disaster Management.

[50] Lu J., Bai Y. Research on low altitude aerial image stitching. Proceedings of the Chinese Control Conference (CCC).

[51] Tsao P., Chen G. Stitching aerial images for vehicle positioning and tracking. Proceedings of the International Conference on Data Mining Workshops (ICDMW).

[52] (1996). ASTM International E1364-95 Standard Test Method for Measuring Road Roughness by Static Level Method 1.

[53] Khoshelham K. Accuracy analysis of Kinect depth data. Proceedings of the ISPRS—International Archives of the Photogrammetry, Remote Sensing and Spatial Information Sciences.

[54] ProVAL (2015). ProVAL User’s Guide.

[55] Karamihas S.M. (2002). Development of cross correlation for objective comparison of profiles. Int. J. Veh. Des..

[56] FHWA-HRT-05-054 Quantification of Smoothness Index Differences Related to Long-Term Pavement Performance Equipment Type. https://www.fhwa.dot.gov/publications/research/infrastructure/pavements/ltpp/05054/chapt4.cfm.

[57] Loizos A., Plati C. (2008). Evolutional process of pavement roughness evaluation benefiting from sensor technology. Int. J. Smart Sens. Intell. Syst..

[58] Gonzalez R., Rodriguez F., Guzman J.L., Pradalier C., Siegwart R. (2012). Combined visual odometry and visual compass for off-road mobile robots localization. Robotica.

